# Quadruple and Truncated MEK3 Mutants Identified from Acute Lymphoblastic Leukemia Promote Degradation and Enhance Proliferation

**DOI:** 10.3390/ijms222212210

**Published:** 2021-11-11

**Authors:** Yoshira M. Ayala-Marin, Alice H. Grant, Georgialina Rodriguez, Robert A. Kirken

**Affiliations:** Border Biomedical Research Center, Department of Biological Sciences, The University of Texas at El Paso, El Paso, TX 79968, USA; ymayala2@gmail.com (Y.M.A.-M.); grantalice6@gmail.com (A.H.G.); grodriguez@utep.edu (G.R.)

**Keywords:** MAP2K3, MEK3, MAPK p38, ALL, Hispanic, protein degradation, leukemia, cell proliferation, health disparities

## Abstract

Compared to other ethnicities, Hispanic children incur the highest rates of leukemia, and most cases are diagnosed as Acute Lymphoblastic Leukemia (ALL). Despite improved treatment and survival for ALL, disproportionate health outcomes in Hispanics persist. Thus, it is essential to identify oncogenic mutations within this demographic to aid in the development of new strategies to diagnose and treat ALL. Using whole-exome sequencing, five single nucleotide polymorphisms within mitogen-activated protein kinase 3 (MAP2K3) were identified in an ALL cancer patient library from the U.S./Mexico border. MAP2K3 R26T and P11T are located near the substrate-binding site, while R65L and R67W localized to the kinase domain. Truncated-MAP2K3 mutant Q73* was also identified. Transfection in HEK293 cells showed that the quadruple-MEK3 mutant (4M-MEK3) impacted protein stability, inducing degradation and reducing expression. The expression of 4M-MEK3 could be rescued by cysteine/serine protease inhibition, and proteasomal degradation of truncated-MEK3 occurred in a ubiquitin-independent manner. MEK3 mutants displayed reduced auto-phosphorylation and enzymatic activity, as seen by decreases in p38 phosphorylation. Furthermore, uncoupling of the MEK3/p38 signaling pathway resulted in less suppressive activity on HEK293 cell viability. Thus, disruption of MEK3 activation may promote proliferative signals in ALL. These findings suggest that MEK3 represents a potential therapeutic target for treating ALL.

## 1. Introduction

Acute lymphocytic leukemia (ALL) is a blood and bone marrow cancer that primarily affects children [1]. It is responsible for causing more deaths than any other childhood cancer, and its incidence is highest among Hispanics [2,3,4]. Less than 1% of biorepository specimens and 2% of samples in genome-wide association studies are derived from Hispanics [5]. This underrepresentation might reflect the disproportionate health outcomes observed in some minority groups [6]. While current ALL treatments have improved the five-year free survival rate to ~90%, not everyone has benefited equally [7]. Some patients experience refractory or ALL relapse, where the 5-year free survival rate falls by between 15 and 50% [8]. Unfortunately, the cause of these statistics is poorly understood due to the complex etiology of ALL. Advances in next generation sequencing support the role of genetic factors in driving ALL. Here, we sought to identify novel mutations from Hispanic patients residing at the U.S/Mexico border that experience a higher incidence of ALL [6]. Understanding the molecular mechanisms responsible for driving ALL in this demographic is essential to developing new therapeutic strategies [9]. 

Using whole-exome sequencing (WES), five single nucleotide polymorphisms (SNPs) on MAP2K3 were identified. MAP2K3 encodes the mitogen-activated protein kinase 3 (MEK3) belonging to the mitogen-activated protein kinase (MAPK) signaling pathway. Dysregulation of the MAPK pathway is frequently observed in various cancers [10]. MEK3 is a dual-specificity kinase that belongs to the MAPKK family responsible for the phosphorylation of serine/threonine and tyrosine residues of downstream MAPK substrates [11]. Critical to the MAPK p38 signaling pathway, MEK3 and MEK6 activate p38 to induce cellular differentiation, migration, survival, apoptosis, and metabolism [12,13,14]. Mice lacking MEK3 and MEK6 are more likely to develop cancer than their wild-type counterparts [15]. Similarly, MEK3 expression is often downregulated in breast, colon, liver, esophageal squamous cell carcinoma, and thyroid cancer [16,17,18,19]. Furthermore, inhibition of p38 is associated with resistance to therapy in hematopoietic malignancies [20,21,22,23]. 

In this study, we investigated the role of four MEK3 mutations found within an ALL cohort, including R65L and R67W localized near the ATP binding site and P11T and R26T residing in the amino-terminal domain [24]. We speculated that MEK3 R65L and R67W would create physicochemical distortions by replacing polar Arg with hydrophobic residues, while P11T and R26T would affect protein binding interactions. Here, we provide evidence that these variants impact MEK3 stability by increasing degradation. Furthermore, MEK3 mutants disrupted auto-phosphorylation, which resulted in a loss of kinase activity and subsequent p38 phosphorylation. MEK3 is responsible for activating MAPK p38 to mediate growth-inhibitory and pro-apoptotic signals. Thus, inhibition of p38 by degradation of MEK3 may render this pathway inactive and contribute to aberrant growth and survival signals in ALL. These findings suggest that MEK3 activation represents a potential therapeutic target for treating ALL.

## 2. Results

### 2.1. Identification of Potential Oncogenes Involved in ALL 

**Figure 1:**
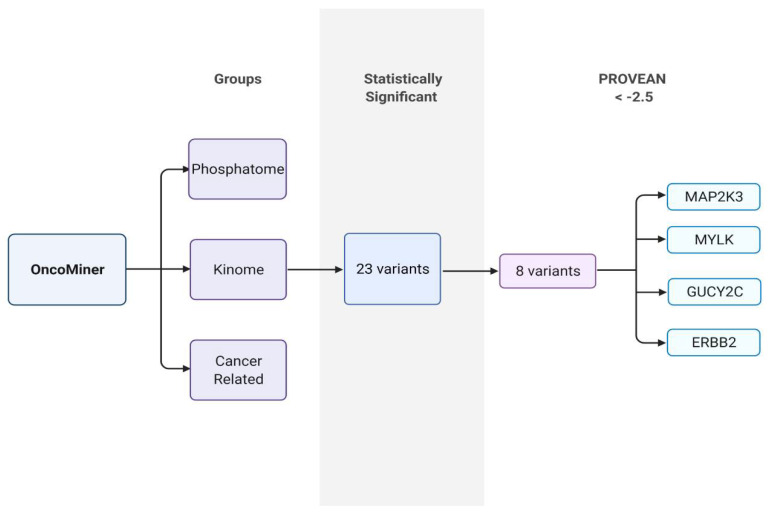
Identification of potential oncogenes involved in ALL. Schematic diagram showing the criteria used to identify ALL variants with a PROVEAN score of less than −2.5.

WES analyzed with OncoMiner Pipeline [25] was used to identify potential genes involved in driving ALL in Hispanic patients from the U.S./Mexico border. OncoMiner variants identified from nine ALL patient samples and seven healthy controls were separated into three groups: kinases, phosphatases, and cancer-related genes. Twenty-three kinase variants observed in ALL were statistically significant from the controls. From these variants, eight were predicted to have a deleterious impact on protein function as indicated by the Protein Effect Analyzer (Provean) score. Five variants occurred in MAP2K3, while the other three occurred in MYLK, GUCY2C, and ERBB2 (Figure 1). The latter variants were confined to amino (MYLK and GUCY2C) or carboxylic domains (ERBB2) and were previously reported as natural variants [26], resulting in their exclusion from subsequent analysis. 

### 2.2. Identification of MEK3 Variants in ALL Patient Samples

**Figure 2:**
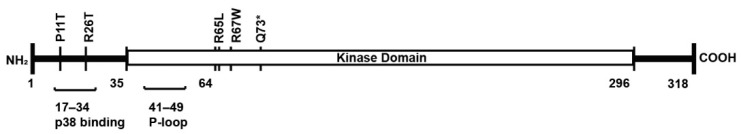
Linear representation of MEK3 indicating location of ALL mutations. Schematic diagram of MEK3 showing mutations identified from the ALL patient library. Amino acids are shown located within structural domains of MEK3.

MAP2K3 isoform-1 was found harboring five potentially pathogenic SNPs indicated by Provean prediction scores of less than −2.5 [27]. Each SNP found in MAP2K3 is shown with its Provean score, exact location, amino acid (A.A.) change, and prevalence in Table 1. Each of them was absent in the control group (Table 1). MAP2K3, R26T, and P11T were located in the amino-terminal end near the p38 binding site, while R65L, R67W, and Q73* localized to the kinase domain near the ATP binding site (Figure 2). Of these, four were previously reported in ClinVar P11T, R26T, R65L, and R67W (dbSNP:rs33911218, dbSNP:rs36047035, dbSNP:rs56067280, and dbSNP:rs56216806, respectively) yet have not been mechanistically characterized. Eight patients harbored the four missense mutations and the unreported nonsense mutation that translates to a truncated MEK3 protein lacking 245 A.A. from its carboxyl-terminal end (Table 1 and Figure 2). Only one patient from the cohort had full-length MEK3 with the four missense mutations near the ATP binding site and the substrate-binding site (Table 1 and Figure 2).

### 2.3. Quadruple and Truncated MEK3 Mutants Decrease Protein Stability

**Figure 3:**
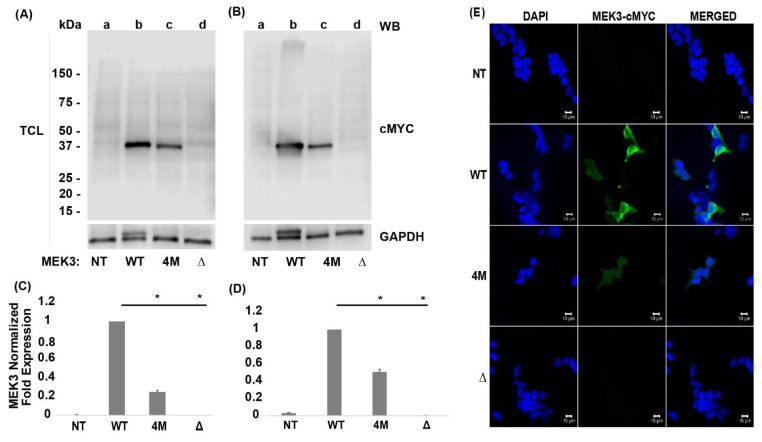
Quadruple and truncated MEK3 mutants decrease protein stability. Cells were not transfected (NT) or transiently transfected concurrently with WT-cMYC or MEK3-cMYC mutant constructs. (**A**) Total cell lysates (TCL) from HEK293 (**B**) and HEPG2 cells transfected with either WT, quadruple (4M), or truncated (Δ) MEK3 constructs, separated by SDS-PAGE (4–20%) and immunoblotted (WB) as indicated. MEK3 protein expression band intensities were normalized to GAPDH using densitometric analysis. (**C**) Quantification of MEK3 mutant protein expression relative to WT in HEK293 and (**D**) in HEPG2 cells. Data are presented as mean ± SEM (*n* = 3). One-way ANOVA with post hoc Tukey’s test for multiple comparisons was used to determine statistical significance * *p* < 0.01. (**E**) Immunofluorescence of transfected HEK293 cells. Subcellular localization of MEK3 was determined by staining for cMYC (middle panel), and nuclei using DAPI (left panel). Right panel shows merged images. Scale bar 20 μM.

To investigate the functional role of MEK3 mutants, quadruple (4M-MEK3) and truncated (Δ-MEK3) MEK3 mutants were generated. Their impact on protein expression was assessed in HEK293 cells transfected with either wild type (WT) or mutant MEK3 constructs. At 24 hours post-transfection, cells were harvested, and total cell lysate (TCL) was probed for MEK3 using an anti-cMYC tag. The 4M-MEK3 construct displayed significantly lower protein expression compared to WT-MEK3 (Figure 3A lanes c and b, respectively). No band was observed for the truncated Δ-MEK3 construct with the expected size of ~15 kDa (Figure 3A lane d). To determine whether the decreased expression of MEK3 was specific to the HEK293 system, HepG2 cells were also transfected with MEK3 constructs. Similarly, 4M-MEK3 had significantly reduced levels of expression compared to WT-MEK3, and again Δ-MEK3 was absent (Figure 3B lanes c and d, respectively). All membranes were reprobed for GAPDH to ensure equal loading (Figure 3C,D). 

Next, dual-labeled immunofluorescent confocal microscopy was utilized to determine whether WT-MEK3 and MEK3 mutants have similar subcellular localization as previously reported [28]. Transfected HEK293 cells were probed for nuclei using the DNA binding fluorescent stain DAPI (Figure 3E, left panel). WT-MEK3 localized to the nucleus and cytoplasm (Figure 3E, right panel) with a punctuate staining pattern in the cytoplasm (Figure 3E, middle panel), while 4M-MEK3 had an equal distribution across the cytoplasm and nucleus. However, the expression of 4M-MEK3 was much lower than WT-MEK3 (Figure 3E, middle panel). There were no detectable levels of Δ-MEK3 (Figure 3E, middle panel), as seen with immunoblot analysis. Taken together, these data suggest that ALL-derived MEK3 mutants not only impact protein expression but also localization.

### 2.4. Quadruple MEK3 Mutant Was Degraded at an Accelerated Rate Compared to Wild-Type Protein

**Figure 4:**
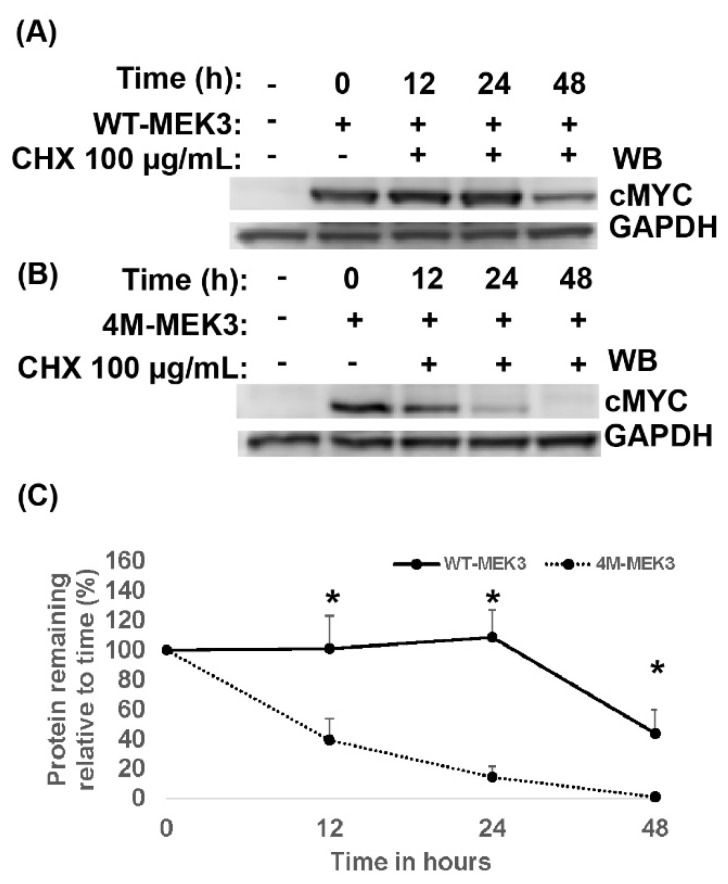
Quadruple MEK3 mutant was degraded at an accelerated rate compared to wild type. HEK293 cells were not transfected (−) or transiently transfected with (**A**) WT-cMYC or (**B**) 4M-MEK3-cMYC mutant construct. Twenty-four hours post-transfection, cells were left untreated (−) or treated with CHX (+) for the indicated time points. Cell lysates were collected and separated by SDS-PAGE and immunoblotted (WB), as indicated. (**C**) Quantified protein expression of WT-MEK3 or 4M-MEK3 is shown relative to time 0. Band intensities were normalized to GAPDH using densitometric analysis and presented as a ratio of cMYC to GAPDH. Data are presented as mean ± SEM (*n* = 3). A t-test (unpaired) was used to determine statistical significance. *, *p* < 0.05.

The observed decreased expression of 4M-MEK3 could be attributed to a higher rate of turnover. Therefore, the half-life of 4M-MEK3 was analyzed in the presence of the translational inhibitor cycloheximide (CHX) and compared to WT-MEK3. Both WT-MEK3 and 4M-MEK3 showed a progressive decrease in protein expression over time, yet their turnover rate differed remarkably (Figure 4). Pronounced turnover of 4M-MEK3 was observed as indicated by a half-life of less than 12 hours (h) compared to WT-MEK3 protein occurring at 48 h (Figure 4). 

### 2.5. Cysteine/Serine Protease Inhibitors Rescue Quadruple MEK3 Mutant Protein Expression

**Figure 5:**
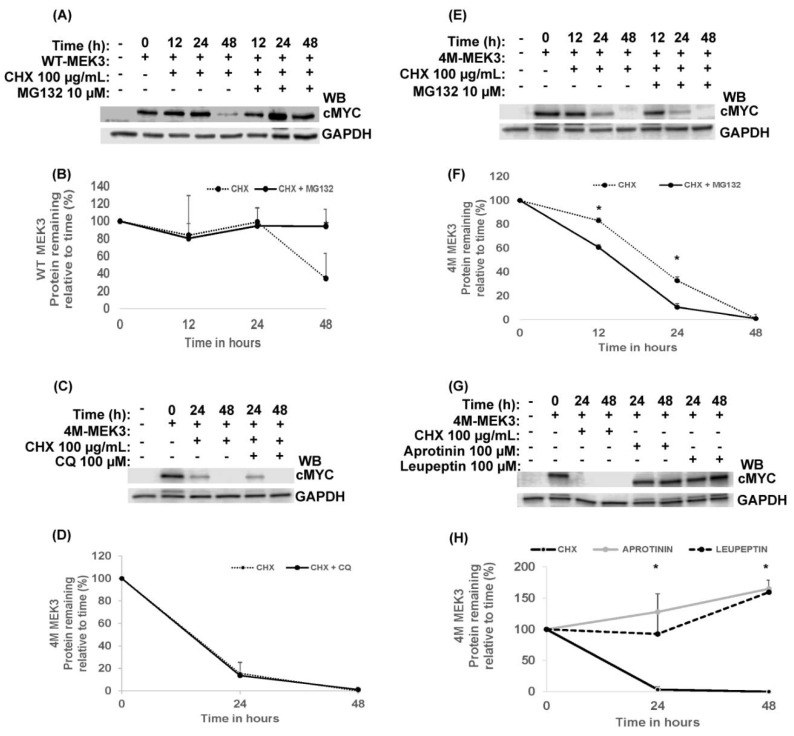
Cysteine/serine protease inhibitors rescue quadruple MEK3 mutant protein expression. HEK293 cells were not transfected (−) or transiently transfected with WT-cMYC or 4M-MEK3-cMYC mutant constructs. At 24 hours post-transfection, cells were left untreated (−) or treated with CHX or a combination with proteolytic inhibitors for the indicated time points. (**A**,**B**) WT-MEK3 was treated with MG132. 4M-MEK3 was treated with either (**C**,**D**) CQ, (**E**,**F**) MG132, or (**G**,**H**) cysteine/serine protease inhibitors. Cell lysates were separated by SDS-PAGE and immunoblotted (WB) as indicated. Quantified protein expression of WT-MEK3 or 4M-MEK3 is shown relative to time 0 (Lower panels **B**, **D**, **F** and **H**). Band intensities were normalized to GAPDH using densitometric analysis and presented as a ratio of cMYC to GAPDH. Data are presented as mean ± SEM (*n* = 3 and *n* = 2 for Panel H). A t-test (unpaired) was used to determine statistical significance. *, *p* < 0.05.

To investigate the proteolytic pathway involved in the degradation of 4M-MEK3, HEK293 cells were transiently transfected with WT-MEK3 or 4M-MEK3 and incubated with CHX in the presence of the following proteolytic inhibitors: MG132 (proteasomal inhibition), chloroquine (CQ) (lysosomotropic agent), and cysteine/serine protease inhibitors (leupeptin and aprotinin, respectively). Expression of WT-MEK3 was stabilized by MG132 proteasomal inhibition (Figure 5A), and no effect was observed in the presence of CQ and the serine/cysteine protease inhibitors (data not shown). Degradation of 4M-MEK3 was accelerated by MG132 proteasomal inhibition, but was insensitive to CQ lysosomotropic agents (Figure 5F,D, respectively). The accelerated degradation observed during proteasome inhibition could suggest that proteins involved in 4M-MEK3 degradation accumulate to facilitate its breakdown. Interestingly, the expression of 4M-MEK3 was rescued by the cysteine/serine proteolytic inhibitors leupeptin and aprotinin (Figure 5G). 

### 2.6. Proteosomal Degradation of Truncated MEK3 Mutant Is Ubiquitin Independent

**Figure 6:**
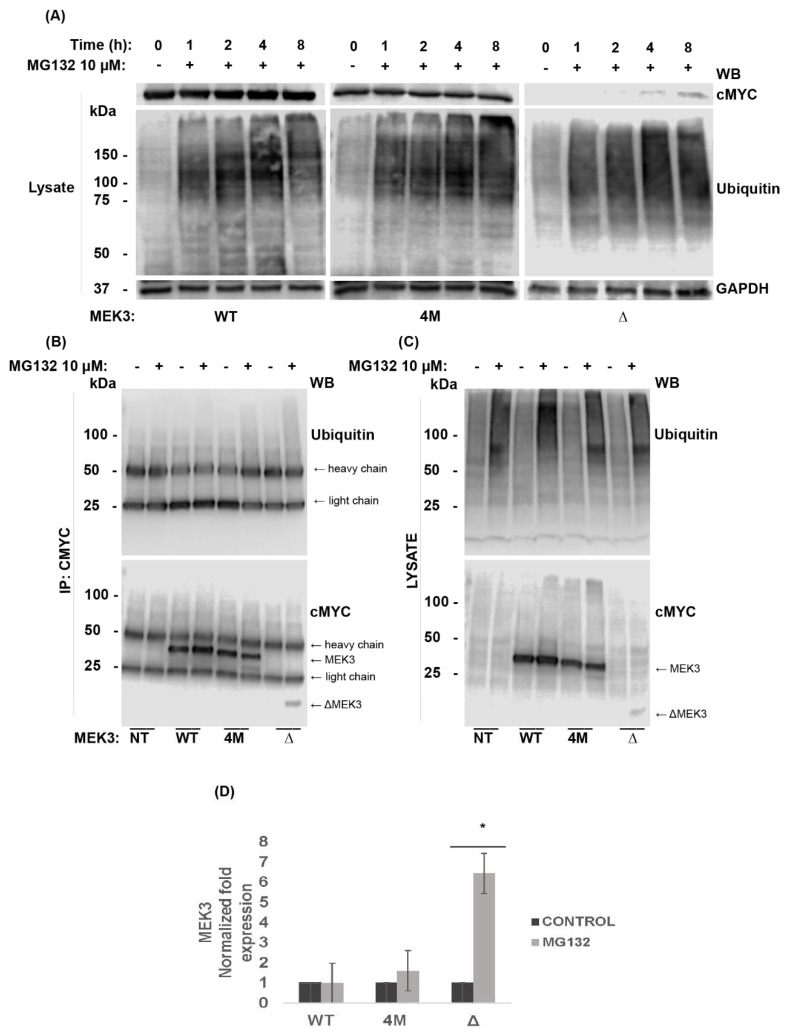
Degradation of truncated MEK3 mutant by the proteasome is ubiquitin-independent**.** HEK293 cells were not transfected (NT) or transiently transfected concurrently with WT-cMYC or MEK3-cMYC constructs (4M-MEK3 and Δ-MEK3). After 24 hours of transfection, cells were left untreated (−) or treated with MG132 (+) as per the indicated time points. (**A**) Cell lysates were separated by SDS-PAGE and immunoblotted as indicated. (**B**) Transfected cells were treated with DMSO (−) or MG132 (+) for 8 h and immunoprecipitated (IP) for cMYC, and (**C**) probed using cell lysate. Samples were separated by SDS-PAGE and immunoblotted (WB) as indicated. (**D**) Quantified protein expression of WT-MEK3 or MEK3 constructs is shown relative to time 0. Band intensities were normalized to GAPDH using densitometric analysis and presented as a ratio of cMYC to GAPDH. Data are presented as mean ± SEM (*n* = 3). T-test (unpaired) was used to determine statistical significance. *, *p* < 0.05.

To explore whether the absence of Δ-MEK3 was due to reduced expression or rapid degradation, HEK293 cells were transfected with MEK3 constructs and incubated without or with MG132. Stable levels of WT-MEK3 and 4M-MEK3 expression were accomplished at 8 h, while the proteasome inhibitor MG132 rescued truncated Δ-MEK3 expression at 4 h (Figure 6A, top panel). These data suggest that Δ-MEK3 is more susceptible to degradation than both WT-MEK3 and 4M-MEK3. As expected, the anti-ubiquitin immunoblot showed an accumulation of ubiquitinated proteins after MG132 treatment (Figure 6A, middle panel). Membranes were reprobed for GAPDH to ensure equal protein loading (Figure 6A, bottom panel). Typically, proteasome degradation requires substrate modification by the addition of ubiquitin to Lys residues. Hence, to assess MEK3 ubiquitination, lysate harvested from transfected cells were immunoprecipitated for c-MYC tagged MEK3, and membranes were probed for ubiquitin. No evidence of ubiquitination was observed (Figure 6B, top panel). Cell lysates from transfected cells probed with anti-ubiquitin showed high-molecular weight ubiquitinated proteins (Figure 6C, top panel). Indeed, several proteins are degraded in a ubiquitin-independent manner, as reviewed by Jariel-Encontre et al., 2008 [29]. The absence of ubiquitination could likely be due to exhaustion of the ubiquitin pool or be due to unstructured regions acting as proteasome initiation sites [29]. The relevance of ubiquitination to MEK3 signaling requires additional study. The absence of detectable Δ-MEK3 is likely due, in part, to enhanced proteasomal degradation. 

### 2.7. MEK3 Mutants Disrupt Auto-Phosphorylation at T222 and Reduce T180/Y182 Phosphorylation of p38 MAPK 

**Figure 7:**
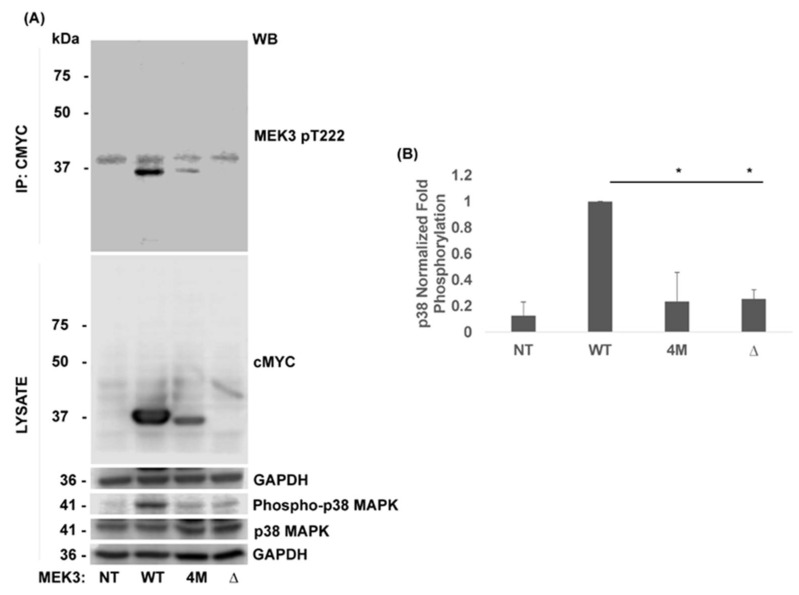
MEK3 mutants disrupt auto-phosphorylation at T222 and reduce T180/Y182 phosphorylation of p38 MAPK. HEK293 cells were not transfected (NT) or transiently transfected concurrently with WT-MEK3 or MEK3-cMYC constructs (4M-MEK3 and Δ-MEK3). (**A**) Immunoprecipitated (IP) cMYC or cell lysate was separated by SDS-PAGE and immunoblotted (WB) as indicated. (**B**) Phospho-p38 band intensities were normalized to total p38 by densitometric analysis. Data are presented as a ratio of cMYC to GAPDH. Quantification of phospho-p38 is shown relative to WT. Representative data for *n* = 3 are presented as mean ± SEM. One-way ANOVA with post hoc Tukey’s test for multiple comparisons was used to determine statistical significance. *, *p* < 0.05.

To determine whether 4M-MEK3 and the newly identified Δ-MEK3 mutant could regulate the MAPK p38 signaling pathway, activation of downstream p38 was assessed. HEK293 cells were transfected with WT-MEK3 or mutant MEK3 constructs. Twenty-four hours post-transfection, cells were lysed, pelleted, and immunoprecipitated for MEK3 using the cMYC antibody. MEK3 autophosphorylation was assessed using a phospho-specific antibody against T222 (pT222). The corresponding lysate was collected and probed for p38 activation using a phospho-specific antibody against MAPK p38 T180/Y182 (Phospho-p38 MAPK). A densitometric analysis of phosphorylated protein was performed and normalized to total cMYC or p38. Compared to WT-MEK3, quadruple 4M-MEK3 and truncated Δ-MEK3 mutants displayed an inability to autophosphorylate (Figure 7A, top panel) and activate p38 (Figure 7A).

### 2.8. Quadruple and Truncated MEK3 Mutants Exhibited Less Suppresive Activity on the Viability of HEK293 Cells

**Figure 8:**
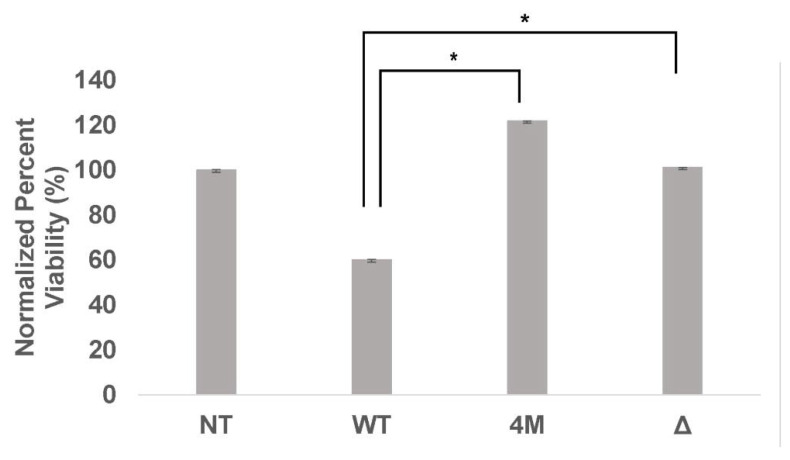
Quadruple and truncated MEK3 mutants exhibited less suppressive activity on the viability of HEK293 cells. HEK293 cells were non-transfected (NT) or transiently transfected concurrently with WT-cMYC or MEK3-cMYC mutant constructs (4M-MEK3 and Δ-MEK3). Cell viability was measured at 24 h using MTS. Values from two independent experiments are presented as percent viability (mean ± SEM). One-way ANOVA with post hoc Tukey’s test for multiple comparisons was used to determine statistical significance *, *p* < 0.01.

Previous studies demonstrated an essential role for MEK3 in cell proliferation by activating p38. Thus, the ability of MEK3 mutants to induce cell proliferation was assessed by MTS assay in transfected HEK293 cells. Cells were seeded in 96-well plates and measured for viability 24 h post-transfection. Data are presented as percent viability. Compared to WT, the quadruple (4M-MEK3) and truncated (Δ-MEK3) mutants showed significantly less suppressive activity on the viability of HEK293 cells (Figure 8). 

## 3. Discussion

ALL represents a significant problem in pediatric health that is marked among Hispanics. Screening for common genetic signatures is becoming standard in diagnostic routines and may exclude oncogenic variants found in Hispanic patients where data have generally been limited. Thus, genomic analyis of diverse cancer patient samples is needed to identfy potential oncogenes and tumor suppressor genes driving ALL. Kinase activating mutations have gained attention given their targetability. However, loss-of-function kinase mutations are also of great importance [30], considering that they can impact protein production, decrease stability or affect folding kinetics. Such mutations can instigate protein unfolding or create incorrectly folded proteins [31]. Here, we present evidence that MEK3 R26T, P11T, R65L, R67W, and Q73* mutations cause instability and limit cellular protein expression (Figure 3). Quadruple 4M-MEK3 expression was rescued by cysteine/serine protease inhibitors (Figure 5). In contrast, the expression of truncated Δ-MEK3 could be rescued by proteasome inhibition, and its degradation was observed to occur in a ubiquitin-independent manner (Figure 6). Furthermore, both quadruple and truncated MEK3 mutants displayed reduced enzymatic activity (Figure 7). Consequently, loss of p38 activation resulted in reduced suppressive activity on HEK293 cell viability. (Figure 8). These data suggest that 4M-MEK3 and Δ-MEK3 mutations prevent auto-phosphorylation, inhibit p38 activation, and promote proliferative signaling within the HEK293 system. It is tempting to speculate a similar role for MEK3 in lymphocytes and ALL. Further studies on MEK3 loss of function mutations and transformation potential are needed to assess MEK3 signaling as a viable target for ALL and other cancers. Here, we show that reoccurring quadruple and truncated MEK3 mutants observed from ALL patients can impact protein expression and downstream MAPK p38 signaling. 

The structural features and the molecular mechanisms implicated in MEK3 turnover have not been characterized. Structural data of MEK family proteins are available for MEK1/2 and MEK6 [32]. MEK3 shares roughly 80% of its homology with MEK6 [28], and most differences are restricted to the amino-terminal end. Residue K64 is essential for kinase activity following binding to the α and β phosphates of ATP [24]. Amino acids R64, R65, and R67 are located within a hydrophobic cluster domain [24]. Unsurprisingly, we discovered that variants within these regions affect both auto-phosphorylation and MEK3 expression (Figure 2 and Figure 7, respectively). It is likely that the quadruple MEK3 mutant is only partially folded compared to WT, resulting in accelerated degradation [33]. These mutations also likely create changes in hydrophobicity and thermal stability. 

The MAPK p38 signaling pathway plays a crucial role in inhibiting cell proliferation and apoptosis [34]. MEK3 arrests growth by upregulating cyclin-dependent kinase (CDK) inhibitors and downregulating both cyclin D1 protein and polycomb protein Bim1 [35,36,37]. It is therefore intuitive that dysregulation of these physiological processes can lead to aberrant consequences and contribute to hematopoietic malignancies. The evidence provided herein supports the hypothesis that the disruption of the MAPK p38 pathway by loss of MEK3 contributes to aberrant cell proliferation (Figure 8). Indeed, the phosphorylation of the retinoblastoma (RB) tumor suppressor by p38 delays G1 cell cycle progression [38]. Conceivably, the inactivation of genes in the MAPK p38 pathway represents one mechanism to escape cell cycle arrest in ALL. The role of p38 in hematopoietic cell proliferation was described elsewhere [39]. 

Current evidence suggests that p38 activation sensitized by genotoxic agents, such as cisplatin and gemcitabine [40], is essential to the antileukemic effects of IFNα [23]. Thus, whether these variants affect resistance to chemotherapeutic agents warrants investigation. Perhaps drugs that mimic p38 phosphorylation of downstream targets or use small-molecule inhibitors to target negative regulators of p38 might elicit a therapeutic benefit. The use of cysteine/serine protease inhibitors for re-establishing p38 activation in the presence of 4M-MEK3 requires further investigation. Reactivating the MAPK p38 pathway may represent a therapeutic strategy for ALL that is particularly relevant to patients harboring MEK3 mutations.

## 4. Materials and Methods 

### 4.1. Whole-exome sequencing

All research utilizing human subjects and human-derived cell lines were approved by The University of Texas at the El Paso Institutional Review Board (UTEP IRB, El Paso, TX, USA) committee. All participants gave written informed consent. The genomic DNA from 7 healthy controls and 9 patients diagnosed with ALL (UTEP biorepository, El Paso, TX, USA) was isolated using Purgene Kit A (Qiagen, Germantown, MD, USA) according to the manufacturer’s instructions, purified, and sent to Otogenetics Corp. Atlanta, GA, USA for WES. 

### 4.2. Bioinformatics

Analysis of WES data was performed by the Border Biomedical Research Core (BBRC, UTEP, El Paso, TX, USA) Bioinformatics unit using OncoMiner to separate variants into three groups: kinases, phosphatases, and cancer-related genes. The kinase group was screened for variants that were predicted to impact protein function using a PROVEAN score of less −2.5 [27] and including those not present within the control group [25].

### 4.3. Cell Culture

The human embryonic kidney HEK293 available from ATCC (ATCC CRL-1573, Manassas, VA, USA) and the human liver cancer HEPG2 obtained from ATCC (ATCC HB-8065™ cell lines were maintained in RPMI 1640 medium containing 10 % fetal bovine serum (FBS; Atlanta Biologicals, R&D Systems, Minneapolis, MN, USA), 2 mM L-glutamine (Corning, New York, NY, USA), and 1 % penicillin-streptomycin (Corning). 

### 4.4. Plasmid and Site-Directed Mutagenesis

The RC218101 plasmid for MAP2K3 was purchased from OriGene (Rockville, MD, USA). The primers were designed using the Agilent mutagenesis primer design tool. Primers included MAP2K3 R65L (forward 5’-tggccgtgaagctgatccgggccac-3’ and reverse 5’-gtggcccggatcagcttcacggcca-3’); P11T (forward 5’-gcacccaaccccacaaccccccgg-3’ and reverse 5’-ccggggggttgtggggttgggtgc-3’); R67W (forward 5’-cgtgaagcggatctgggccaccgtgaa-3’ and reverse 5’-ttcacggtggcccagatccgcttcacg-3’); R26T (forward 5’-ttcatcaccattggagacactaactttgaggtggaggctg-3’ and reverse 5’-cagcctccacctcaaagttagtgtctccaatggtgatgaa-3’); Q73 stop (forward 5’-ccgggccaccgtgaactcataggagcagaa-3’ and reverse 5’-ttctgctcctatgagttcacggtggcccgg-3’). MEK3 mutants were made using the Quickchange II XL Site-directed Mutagenesis kit (Agilent Technologies, Santa Clara, CA, USA) according to the manufacturer’s instructions. The MEK3 quadruple mutant (4M-MEK3) contained R65L, P11T, R67W, and R26T mutations, whereas the truncated MEK3 (Δ-MEK3) contained the Q73 stop mutation. All mutations were verified by DNA sequencing at the Genomic Analysis Core Facility of the BBRC at UTEP, El Paso, TX, USA.

### 4.5. Transfection, Lysis of Cells, Immunoprecipitation, and Western Blots

HEK293 cells were seeded in 6-well plates and transiently transfected until they reached 90–95% confluency. Transfection efficiency was measured by the expression of the target protein at different time points (i.e., 24, 36, and 48 hours). Maximum protein expression occurred at 24 h and was utilized for all transfection experiments. MEK3-WT, 4M-MEK3, or Δ-MEK3 were transfected (3 µg) concurrently using 5 µl of Invitrogen Lipofectamine 2000 (Thermo Fisher Scientific, Waltham, MA, USA) according to the manufacturer’s instructions. At 24 hours post-transfection, cells were pelleted and lysed in Triton lysis buffer, as previously described [41]. Total protein concentration was determined using the Pierce bicinchoninic acid method (Thermo Fisher Scientific, Waltham, MA, USA). MEK3 constructs were immunoprecipitated using 2 µg of the cMYC antibody (SC-40). Samples were separated in 4–20% SDS-PAGE, transferred to a PVDF membrane, and immunoblotted as previously described [41] using the following antibodies: MEK3 pT222 (OriGene, Rockville, MD, USA) or p38 ppT180/Y182 (cell signaling 4115S). Anti-cMYC (SC-40) was used to assess total MEK3; anti-ubiquitin (A104R) was used to detect protein ubiquitination; and anti-GAPDH was used as a loading control. Membranes were developed using HRP goat anti-rabbit or anti-mouse antibodies and visualized by enhanced chemiluminescence using LICOR and Image Studio Lite software (Lincoln, NE, USA). For reblotting, membranes were placed in the stripping buffer (62.5 mM Tris (pH 6.7), 2% SDS, and 100 mM β-mercaptoethanol) for 30 min at 55 °C, then blocked and reprobed with anti-GAPDH or anti-cMYC. 

### 4.6. Immunofluorescence 

Transfected HEK293 cells were seeded in a 96-well plate, fixed with 8% formaldehyde and permeabilized with 0.1% Tween 20 v/v in PBS. Cell staining was performed as previously described [42] using the mouse monoclonal anti-cMYC (Santa Cruz Biotechnology, Dallas, TX, USA) and goat anti-mouse IgG (H+L) Alexa Fluor Plus 488 as a secondary antibody. High-resolution digital fluorescent images were captured from the stained cells using a LSM 700 confocal laser scanning microscope equipped with a 40× immersion oil objective (Zeiss, New York, NY, USA). Images were captured in the multitrack scanning mode (Alexa-488 and DAPI) and analyzed using the ZEN 2009 software (Zeiss). Visualization of transfected cells by fluorescence microscopy against the cMYC tag was used to estimate the transfection efficiency of MEK3. Transfection efficiency was determined as follows: number of cells stained with fluorescent dye divided by the total number of cells in the field, and this figure was multiplied by 100. This method yielded a transfection efficiency of roughly 75% in the HEK393 model system.

### 4.7. Protein Half-Life Studies 

HEK293 cells were plated in 6-well plates and transfected as previously stated. At 24 h post-transfection, cells were incubated without or with the translational inhibitor cycloheximide (CHX) at a final concentration of 100 µg/mL. Cells were collected at 0, 12, 24, and 48 hours. Similarly, to investigate protein turnover, cells were treated with CHX with the addition of proteolytic inhibitors: proteasome inhibitor MG132 (10 μM), lysosomotropic agent chloroquine (CQ) (100 μM), or the cysteine/serine protease inhibitors (aprotinin and leupeptin) (100 μM). Non-transfected (NT) cells were used as a negative control.

### 4.8. Cell Viability Assay

To examine whether MEK3 mutants impact proliferation, HEK293 cells were transfected in 6-well plates with 3 μg of WT or MEK3 mutant plasmids. At 24 h post-transfection, cells were harvested and plated in 96-well plates and incubated overnight at 37 °C, allowing cells to become adherent. Cell viability was measured using the MTS (3-(4,5-dimethylthiazol-2-yl)-5-(3-carboxymethoxyphenyl)-2-(4-sulfophenyl)-2H-tetrazolium) reagent (Promega, Madison, WI, USA) as previously described [43]. Absorbance was measured using a iMark microplate reader (BIO-RAD, Hercules, CA, USA). Data represent the mean and standard deviations from two independent experiments in triplicates and are presented as percentages of cell viability.

## Figures and Tables

**Table 1:** Identification of SNPs in the MAP2K3 gene using the OncoMiner Pipeline.

MAP2K3	Provean	A.A.From	Location	A.A.To	Patients	Controls
chr17.21202191.c.a.c	−5.539	P	11	T	9	0
chr17.21202237.g.c.g	−2.605	R	26	T	9	0
chr17.21204187.g.t.g	−6.557	R	65	L	9	0
chr17.21204192.c.t.c	−5.085	R	67	W	9	0
chr17.21204210.c.t.c	−7.739	Q	73	*	8	0

* Stop codon.
